# Scoring Systems Estimating Length of Stay in Intensive Care Unit Before Coronary Artery Bypass Grafting

**DOI:** 10.21470/1678-9741-2021-0131

**Published:** 2022

**Authors:** Fatih Aksoy

**Affiliations:** 1 Department of Cardiology, Faculty of Medicine, Suleyman Demirel University, Isparta, Turkey. E-mail:

We interestingly read the article by Zarrizi et al.^[1]^ entitled “Predictors of Length of Stay in Intensive Care Unit after Coronary Artery Bypass Grafting”. First, I congratulate the authors for their invaluable contribution to the literature. On the other hand, I would like to point out some information about the scores and preoperative estimating in intensive care stay.

The authors in that article developed a scoring system including number of chest tubes, development of atrial fibrillation, and atelectasis. Accordingly, they showed that this scoring system was useful in predicting the length of stay in intensive care unit after coronary artery bypass grafting. Although the results of this study were useful for clinical practice, they do not provide additional information on the duration of intensive care unit length of stay before coronary artery bypass grafting. In a previous article, we showed that the CHA2DS2-VASc - congestive heart failure, hypertension, age ≥ 75 years, diabetes mellitus, stroke or transient ischemic attack, vascular disease, age 65 to 74 years, sex category - score correlates with coronary care unit length of stay and new-onset atrial fibrillation in patients with ST elevation myocardial infarction^[2]^. Additionally, we showed that the CHA2DS2-VASc and anticoagulation and risk factors in atrial fibrillation (ATRIA) scores were useful in detecting postoperative atrial fibrillation after coronary artery bypass grafting and that these scores are related to intensive care unit length of stay^[3]^. I believe that it would be useful if the authors provided data about these easy scoring systems on intensive care unit length of stay.

The CHA2DS2-VASc and ATRIA risk scores are cheap and easy scoring systems that are used to predict the risk of thromboembolism in non-valvular atrial fibrillation patients^[4^,^5]^. It has been showed that these scores predicted several anatomical and clinical diseases in cardiovascular practice^[6^-^9]^. Because of that, these scoring systems may be used to assess risk and estimate length of stay in intensive care in patients undergoing coronary artery bypass grafting.
